# To Delinquents

**Published:** 1857-01

**Authors:** 


### "TO DELINQUENTS."

"A number of persons, here and there, have regularly received this Journal through the year, until about the tenth or eleventh number, and then either send us a single number marked "*Refused,*" or have the P. M. do so for them. We shall not forget these 'high-toned' gentlemen."—*Cincinnati Med. Observer.*

Some such experience, we are sorry to say, (not so much on our own account,) has fallen to our lot; we are perfectly willing, and expect, to suffer loss in many ways connected with the publication of a journal, but it is always unpleasant to see manifestations of this character in a profession, all the members of which should be gentlemen; *we* shall not fail to recollect a few individuals of this class.

